# Mitochondrial DNA Variant Discovery and Evaluation in Human Cardiomyopathies through Next-Generation Sequencing

**DOI:** 10.1371/journal.pone.0012295

**Published:** 2010-08-20

**Authors:** Michael V. Zaragoza, Joseph Fass, Marta Diegoli, Dawei Lin, Eloisa Arbustini

**Affiliations:** 1 Genetics & Metabolism Division, Pediatrics Department and Center for Mitochondrial and Molecular Medicine and Genetics (MAMMAG), University of California Irvine, Irvine, California, United States of America; 2 Bioinformatics Core, UC Davis Genome Center, University of California Davis, Davis, California, United States of America; 3 Centre for Inherited Cardiovascular Diseases, IRCCS Foundation Policlinico San Mateo, Pavia, Italy; Duke University Medical Center, United States of America

## Abstract

Mutations in mitochondrial DNA (mtDNA) may cause maternally-inherited cardiomyopathy and heart failure. In homoplasmy all mtDNA copies contain the mutation. In heteroplasmy there is a mixture of normal and mutant copies of mtDNA. The clinical phenotype of an affected individual depends on the type of genetic defect and the ratios of mutant and normal mtDNA in affected tissues. We aimed at determining the sensitivity of next-generation sequencing compared to Sanger sequencing for mutation detection in patients with mitochondrial cardiomyopathy. We studied 18 patients with mitochondrial cardiomyopathy and two with suspected mitochondrial disease. We “shotgun” sequenced PCR-amplified mtDNA and multiplexed using a single run on Roche's 454 Genome Sequencer. By mapping to the reference sequence, we obtained 1,300× average coverage per case and identified high-confidence variants. By comparing these to >400 mtDNA substitution variants detected by Sanger, we found 98% concordance in variant detection. Simulation studies showed that >95% of the homoplasmic variants were detected at a minimum sequence coverage of 20× while heteroplasmic variants required >200× coverage. Several Sanger “misses” were detected by 454 sequencing. These included the novel heteroplasmic 7501T>C in tRNA serine 1 in a patient with sudden cardiac death. These results support a potential role of next-generation sequencing in the discovery of novel mtDNA variants with heteroplasmy below the level reliably detected with Sanger sequencing. We hope that this will assist in the identification of mtDNA mutations and key genetic determinants for cardiomyopathy and mitochondrial disease.

## Introduction

### Mitochondrial DNA and inheritance

Human mitochondrial DNA (mtDNA) is a circular, 16,569 base sequence that encodes for 13 proteins, 22 transfer RNAs (tRNAs) and 2 ribosomal RNAs [1]. MtDNA is essential for mitochondrial energy production through oxidative phosphorylation, and hundreds to thousands of mtDNA molecules may be found per cell depending upon the energy requirements of the tissue [2].

In contrast to nuclear genes (nDNA), mtDNA is inherited only from the mother [3]; therefore, mtDNA mutations associated with inherited mitochondrial diseases follow the maternal lineage with no transmission from the father [4]. Along the maternal lineage, different amounts of a pathogenic and normal mtDNAs may be inherited due to a genetic bottle neck during oogenesis and/or by purifying selection of severe mtDNA mutations [5]–[7]. Homoplasmy describes the state where only mutant or variant mtDNAs exist; whereas, in heteroplasmy there is a mixture of normal and mutant or variant mtDNAs. Depending on the inheritance (maternally-derived or *de novo*), segregation and postnatal selection, an affected individual may have different ratios of mutant and normal mtDNA in different tissues that result in mitochondrial disease.

### Mitochondrial DNA analysis by capillary and next generation sequencing

Traditionally, the most comprehensive method used to detect variants is Sanger capillary sequencing of whole mtDNA [8], [9]. However, limitations prevent the routine use of whole mtDNA studies by Sanger sequencing including the amount of labor and time to manually inspect data for heteroplasmic mutations. Thus, large scale efforts have been lacking to critically evaluate the role of mtDNA mutations in human disease and to investigate the scope of normal mtDNA variation in populations.

Recently, advances in microfluidics, digital imaging systems and bioinformatics have lead to new sequencing methods or Next Generation Sequencing (NGS) that may overcome these limitations. NGS methods can generate vast amounts of sequencing data in less time and overall costs compared to traditional methods [10]. So, many people believe that NGS may replace the traditional methods to detect pathogenic mutations in patients. However, a major problem is the lack of patient studies to evaluate NGS for mutation detection in comparison to Sanger sequencing [11]. In addition, few NGS studies have focused on mtDNA [12], [13] and the detection of heteroplasmy [14], [15] with only one study on patients with known or suspected mitochondrial disease [16].

The goal of this study was to explore NGS as a method to detect mtDNA mutations in patients. Our aims were: (1) to identify all mtDNA variants and potential mutations; (2) to evaluate variant detection performance by comparing Roche 454 pyrosequencing and Sanger sequencing technologies; and (3) to estimate the amount of sequence coverage needed to detect homoplasmic and heteroplasmic mtDNA mutations or variants. In this report, we provide the results of mtDNA analysis for 20 patients with mitochondrial cardiomyopathy or suspected mitochondrial disease.

## Results

### Study population

We studied the mtDNA for 20 cases: 18 with mitochondrial cardiomyopathy and 2 with suspected mitochondrial disease. For each case, general features and the numbers of mtDNA variants detected by Sanger and 454 sequencing are listed in Table 1.

**Table 1:** Summary of mtDNA mapping and sequencing results for 20 cases.

Case	Cardiac	454 Mapping Ave.^b^		454 Variants^c^				Sanger Variantsa,c			
No.^a^	Feature	Depth	Length	Total	Subst	Indel	Repeat	Total	Subst	Indel	Repeat
**1** d	HCM, HF	463	355	**39**	34	3	2	**37**	33	0	4
**3**	HCM	1,148	377	**23**	14	9	0	**15**	14	0	1
**5**	SCD, HF	944	373	**33**	31	2	0	**32**	30	0	2
**6**	DCM	1,542	374	**28**	17	11	0	**18**	17	0	1
**7**	HCM	1,561	372	**25**	17	8	0	**18**	17	0	1
**8**	HCM	1,294	381	**24**	15	8	1	**18**	15	0	3
**9**	HCM	1,506	374	**20**	12	7	1	**15**	13	0	2
**10**	HCM	1,635	377	**19**	10	8	1	**12**	10	0	2
**11**	HCM	1,393	373	**25**	10	14	1	**14**	10	1	3
**12**	HCM	1,442	379	**17**	10	6	1	**13**	11	0	2
**13**	DCM	1,042	373	**39**	32	6	1	**36**	32	1	3
**14**	DCM	1,498	372	**35**	25	7	3	**28**	25	0	3
**15**	DCM	2,140	376	**35**	25	10	0	**26**	25	0	1
**16**	DCM	1,823	381	**15**	9	6	0	**10**	9	0	1
**17**	DCM	1,134	375	**38**	30	8	0	**30**	29	0	1
**18**	DCM	1,431	369	**45**	36	8	1	**41**	36	1	4
**19**	DCM	1,147	368	**17**	7	9	1	**10**	7	0	3
**20**	DCM	2,054	380	**51**	40	11	0	**41**	39	0	2
**NonCM1**	ECHO nl	57^e^	363	**30**	29	1	0	**31**	29	0	2
**NonCM2**	ECHO nl	716	376	**23**	14	9	0	**15**	13	0	2
	**AVE:**	**1,299**	**373**	**29**	**21**	**8**	**1**	**23**	**21**	**0**	**2**
	**TOTAL:**	**–**	**–**	**581**	**417**	**151**	**13**	**460**	**414**	**3**	**43**

Abbreviations: HCM =  hypertrophic cardiomyopathy; HF =  heart failure; DCM =  dilated cardiomyopathy; SCD =  sudden cardiac death; ECHO nl =  echocardiograms normal.

a Additional case information and Sanger data analysis for haplotype and potential mutations are described in a separate report (unpublished data); cases 2 and 4 were not included in the present study.

b 454 mapping statistics: depth =  average number of mapped sequences (non-redundant and redundant sequences) at each base position; length =  average base length of mapped sequences.

c Mitochondrial DNA variants compared to NCBI Reference (NC_012920); Variant types: subst =  single nucleotide substitutions; indel =  insertion or deletion; repeat =  polymorphic repeats at positions: 303–315, 522–523, 574, 16180–16193 and 16519 (www.mitomap.com).

d From previously reported HCM family with *MYH7* mutation [17].

e Decreased reads for NonCM1 possibly due to a pre-sequencing technical error.

### MtDNA variant calls based on bioinformatics analysis of 454 sequences

For Sanger sequencing, we made 330,979 (99.9%) of 331,380 possible base calls (20 mtDNAs ×16,569 bases/mtDNA). Compared to the reference, we identified 460 mtDNA variants that consisted of 414 single nucleotide substitutions, 410 homoplasmic and 4 heteroplasmic variants (Table 1). For each case, we found 10 to 41 variants for an average of 23 variants per mtDNA; details for the Sanger mtDNA variants will be in a separate report (unpublished data).

For 454 sequencing, the results are provided in Table 1 for total coverage and read length; Table S1 for detailed results for all variants; and Table S2 for the mapping statistics. Using a single 454 run, we obtained approximately 440 Mb of raw sequencing data from 1.2 million sequence reads. On average, 97.1% of the reads and 98.4% of the bases mapped to the reference sequence for each case (Table S2) which corresponded to an average coverage (redundant and non-redundant sequences) of 1,300× and to an average mapped read length of 373 bases (range: 355 to 380 bases) (Table 1).

**Figure 1:**
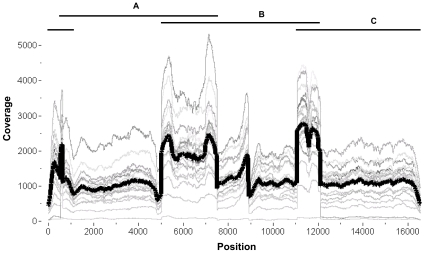
Variability in 454 sequence coverage. Total read coverage (redundant and non-redundant) is plotted at each mtDNA position (1 to 16569) and graphed as a continuous thin gray trace for each case (different shades for each case). The mean coverage for all 20 cases is represented by a thick dark black trace. The shape of the traces shows coverage variability both between cases and along the same mtDNA. The black horizontal lines (A, B & C) above the graph represent the three mtDNA PCR fragments used for 454 sequencing. Greater coverage was noted in the regions in which the PCR fragments overlap compared to coverage in non-overlapping regions.

Similar to previous studies on 454 sequencing, we found variability in the depth of coverage between multiplexed cases and along the same mtDNA molecule (Figure 1) [11], [12], [18]. We attributed the lowest coverage due to a technical error in sample preparation for NonCM1. When this case was excluded, we still found that the read depth varied between cases up to ten-fold (Figure 1). For coverage along the same mtDNA, we observed a significant increase in the average coverage per base within the regions where the PCR fragments overlap (mean = 1,947, SD = 991) compared to the average coverage in non-overlapping regions (mean = 1,072, SD = 543), *p*<0.0001. Overall, these findings suggest that uniform coverage might be achieved by single or two fragment LR-PCR instead of three fragments [12], [19].

We mapped the reads using the Roche GS Mapper software and identified “high confidence” differences (HCDiffs) between each mtDNA contig and the reference (Table 1). For the 20 cases, all 331,380 possible base calls were made. These included 581 HCDiffs with 417 single nucleotide substitutions, 411 homoplasmic and 6 heteroplasmic variants (Table 2 and Table S1). For each case, we found 15 to 51 HCDiffs for an average of 29 variants per mtDNA.

**Table 2:** Discordant and/or heteroplasmic substitution mtDNA variants.

	Method^a^				454 depth		Reason for
Type	Sanger	454	Case No.	Position	(% variant) b	Type of error	discordance
Homoplasmic	+	−	9	8473T>C	231 (83%)	454 false -	Homopolymer
	+	−	12	16566G>A	352(100%)	454 false -	Software miscall
	−	+	17	3010G>A	350 (100%)	Sanger false -	Miscall
	−	+	20	15326A>G	405 (100%)	Sanger false -	Miscall
	−	+	1	13967C>T	244 (100%)	Sanger false -	Poor data
Heteroplasmic	−	+	NonCM2	16093T>C	246 (14%)	Sanger false -	Miscall
	−	+	5	7501T>C	298 (18%)	Sanger false -	Miscall
	+	+	6	3243A>G	340 (19%)	N/A	N/A
	+	+	20	9854T>G	344 (39%)	N/A	N/A
	+	+	7	3645T>C	382 (47%)	N/A	N/A
	+	+	NonCM1	15222A>G	45 (64%)	N/A	N/A

a Sequencing method: variant detected =  “+”, variant not detected =  “−”.

b 454 depth =  total number of non-redundant reads with percentage of variant reads in parentheses.

### Comparison of 454 and Sanger results indicated high concordance for substitutions

**Figure 2:**
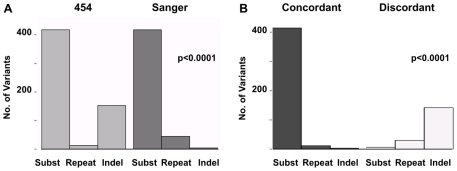
Comparison of 454 and Sanger sequencing results. Histograms show the distribution of variants by type: single nucleotide substitutions (subst), repeats and insertion/deletions (indel). **A. Sequencing method.** Results are separated by detection using 454 (left panel, light gray) or Sanger (right panel, dark gray). **B. Concordance.** Results are separated by concordant detection using 454 and Sanger sequencing (left panel, dark gray) or by discordant detection, 454 or Sanger sequencing only (right panel, light gray). We observed high concordance between the two methods for mtDNA substitutions and significant discordance for indels.

We first compared the numbers and types of variants (substitutions, indels and repeats) detected by 454 and Sanger sequencing (Table 1). There was a significant difference due to a greater number of indels detected by 454, *X* ^2^ (2, N = 1041)  = 146.2, p<0.0001 (Figure 2A). In total, 614 mtDNA variants were identified by 454 or Sanger sequencing (Table S1). These included 427 (70%) concordant variants (detected by both platforms) and 187 (30%) discordant variants (initially detected by a single method). The 427 concordant variants consisted of: 412 of 419 (98%) substitutions, 12 of 44 (27%) repeats and 3 of 151 (2%) indels (Figure 2B). Compared to the concordant variants, the distribution of 187 discordant variants was significantly different and included: 7 of 419 (2%) substitutions, 32 of 44 (73%) repeats and 148 of 151 (98%) indels, *X* ^2^ (2, N = 614)  = 526, p< 0.0001 (Figure 2B).

### Discordant variants include a potential, novel mtDNA mutation missed by Sanger

Next, we wanted to determine the possible sources of error for the seven discordant substitution variants, two (8473T>C and 16566G>A) detected by Sanger and five identified by 454 (Table 2). We first reviewed the Sanger chromatograms and 454 reads that mapped to these positions and concluded that all seven discordant results were false negative errors. For 8473T>C, the discordance may be from masking of variant reads by erroneous 454 reads due to the formation of a six base homopolymer (Table S1). For 16566G>A, we found the variant in all mapped reads at that position; however, it was not identified as a high confidence variant.

**Figure 3:**
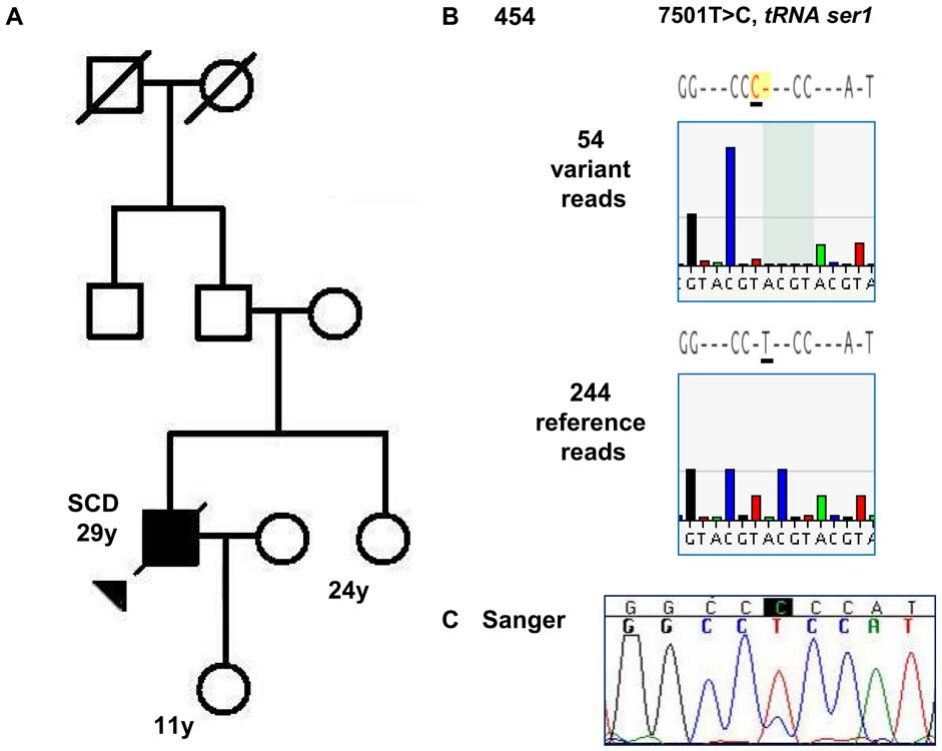
Novel, potential mtDNA mutation 7501T>C detected by 454 sequencing. **A. Case 5 pedigree.** Circles represent females and squares represent males; solid shapes are affected individuals; an arrow indicates the proband that died from sudden cardiac death (SCD). **B. 454 sequencing.** Shown are representative 454 data flowgrams. We obtained 298 total unique reads, 54 reads had the 7501T>C variant (top) and 244 reads had the reference sequence (bottom). **C. Sanger sequencing.** Retrospective analysis of Sanger data revealed the missed variant. As shown in the chromatogram, two peaks are found at position 7501 consistent with a low level of heteroplasmy.

For the five discordant variants detected by 454 (Table 2), retrospective review of the initial chromatograms and repeat Sanger sequencing confirmed the presence of each variant. From this, we concluded that four false negative errors resulted from miscalled Sanger sequences, and the fifth Sanger “miss” to the lack of coverage. We found that the most significant miscall was a heteroplasmic variant, 7501T>C in *MT-TS1* for tRNA serine 1 (Figure 3).

### Analysis of heteroplasmic mtDNA variants

We next compared the results of 454 and Sanger sequencing for the detection of heteroplasmic variants defined as having 80% or less variant read frequency after excluding erroneous reads from a homopolymer. We evaluated all substitution and indel variants detected by 454 and excluded repeat polymorphisms because of the possibility of false positive errors from PCR amplification [20].

Based on these criteria, we found six heteroplasmic variants of 419 total substitutions. These included four of 412 concordant substitutions (3243A<G, 9854T<G, 3645T<C and 15222A<G) with variant read frequencies of 19% to 64% and two of seven discordant substitutions (16093T>C and 7501T>C) with frequencies of 14% and 18% (Table 2). Similar to 7501T>C, we failed to identify 16093T>C by our initial Sanger analysis and detected the variant first by 454 sequencing.

We continued our evaluation to determine the presence of heteroplasmy for the 151 indels detected by 454 sequencing. Based on our criteria, indels had a significantly higher proportion of variants with read frequencies of 80% or less (148 of 151 indels) compared to substitutions (6 of 419), *X* ^2^ (1, N = 570)  = 520, p<0.0001. However, after reviewing all 454 reads for the 148 indels, we found that each indel was associated with a homopolymer (4 to 8 bases) at or within four bases of the variant position (Table S1). So, we concluded that all 148 indels were most likely homopolymer-associated false positive errors and were not true heteroplasmic variants, findings consistent with the Sanger data for these positions.

### Performance metrics for 454 and Sanger sequencing

As another means of comparison, we used performance metrics [11] that we modified to reflect mtDNA genotypes with heteroplasmy (Table S3). We calculated 454 sequence accuracy as 99.95% and variant accuracy as 99.5% excluding repeat polymorphisms. Next, we calculated the error rates and found no significant difference in the false negative rate for 454 (2 of 421 = 0.5%) and Sanger (4 of 421 = 1%), *X* ^2^ (1, N = 842)  = 0.168, p = 0.6820. However, we found a significantly greater false positive rate for 454 (148 of 329938 = 0.044%) compared to Sanger in which we did not find false positive errors (0 of 329938 = 0%), *X* ^2^ (1, N = 659876)  = 148.033, p<0.0001, as we expected from homopolymer-associated 454 indels.

### Simulation studies- variant detection at different coverages

**Figure 4:**
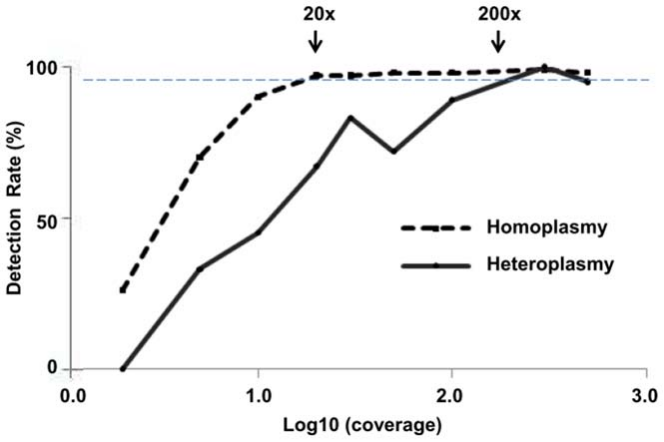
Coverage simulations and variant detection rate for mtDNA variants. By subsampling all mapped 454 reads, we simulated nine levels of coverage (2× to 500×) for 419 mtDNA substitution variants. Graph shows the variant detection rate by the log10 (coverage) for homoplasmy (black solid line, n = 413) and heteroplasmy (black dashed line, n = 6). We estimated that the minimum coverages to detect 95% of the variants (gray dashed horizontal line) are >20× for homoplasmy and >200× for heteroplasmy.

After our comparisons between 454 and Sanger sequencing, we estimated the minimum 454 sequence coverage for detection of heteroplasmy (Figure 4 and Table S4). We simulated nine levels of coverage from 2× to 500× and then calculated the variant detection rate for 419 total substitution variants: 6 heteroplasmic variants and 413 homoplasmic variants. In this analysis, we excluded repeat polymorphisms and indels because nearly all indels were homopolymer-associated false positive errors and the possibility of the repeats being PCR artifacts. From these simulations, we estimated that to detect >95% of mtDNA variants by 454 sequencing, read coverages of at least >20× for homoplasmy and >200× for heteroplasmy (range: 10%–80%) were needed.

## Discussion

### Roche 454 versus Sanger sequencing for whole mtDNA analysis

Our results showed that 454 sequencing was comparable to Sanger sequencing in the detection of single nucleotide substitutions. High concordance (98%) was obtained for the detection of over 400 single nucleotide substitutions including four heteroplasmic variants previously detected by Sanger sequencing. A previous 454 sequencing study of a 110 kb nDNA region supports this finding; the study found 98.5% agreement for nearly 400 SNPs at 30× average coverage [21].

This high concordance, in addition, is reflected in the high 454 sequencing (99.95%) and variant accuracies (99.5%) in our study. The 454 sequencing accuracy is consistent with the NGS study that also directly compared results to Sanger analysis; they obtained >99.9% 454 sequencing accuracy for a 266 kb nDNA region in 4 samples with 43× average coverage [11]. Our variant accuracy, however, was significantly greater than the 454 variant accuracy (93.9%) [11]. Since variant accuracy only includes Sanger detected variants, one possible explanation for this difference was our exclusion of repeat polymorphisms—identified commonly by Sanger but not by 454 sequencing. To confirm these repeats are real variants (and thus, true 454 false negatives), non-amplification and single molecule analysis may be possible using the next wave of sequencing technologies [22].

For our discordant results, not surprisingly, we found 454 indels consistent with false positive errors from homopolymers in previous reports [23]–[25]; however, we found, in addition, two 454 false negative errors that resulted in an ∼0.5% false negative rate. This rate was less than the 3% 454 false negative rate previously reported [11], possibly again due our exclusion of repeat polymorphisms. For SNP calling, previous 454 studies reported no false negatives with average coverages at 30× [21] and 43× [11].

Including the results of our studies, we concluded that false negative errors for SNP calling were rare for 454 sequencing. For one of the 454 false negatives 8473T>C, we concluded it was from the unusual creation of a new homopolymer with the variant. The T>C base change created a six cytosine homopolymer (position 8471–8476) by linking adjacent cytosine bases. The resulting indel reads (frequency = 17%) associated with the homopolymer may have masked the call of 8473T>C as a high confidence variant. The second 454 false negative, 16566G>A, likely resulted from a software error. Although 16566G>A was found in all mapped sequences (352 unique reads) of the contig, 16566G>A was not called as a 454 variant. In support, we found the updated Roche GS Mapper software (version 2.3) correctly called the variant (data not shown).

Taken together, these results show that 454 sequencing was reliable as Sanger sequencing in the SNP detection. Still, one concern may be the decreased ability to resolve homopolymeric regions by 454 sequencing that potentially may mask a mutation at or adjacent to the region. It is possible that this may have little impact on the ability to detect mtDNA mutations. We found that the 148 homopolymer-associated indels involved only 23 nucleotide positions (Table S1). The vast majority were located in coding regions; however, mutations associated with mitochondrial disease have not been reported at any of these positions (www.MITOMAP.org).

### Detection of missed heteroplasmy by 454 sequencing

With the remaining discordant results, we provide evidence that 454 sequencing may detect variants missed by Sanger analysis—mtDNA variants with roughly less than 20% heteroplasmy. We first noted, overall, five Sanger false negatives that resulted in a 1% Sanger false negative rate. Our estimated rate was less than the 3% Sanger false negative rate previously reported [11]; we speculate this difference may be from improved variant detection by duplicate mtDNA analyses in two laboratories. Still, we had five Sanger false negatives including two mtDNA variants with low levels of heteroplasmy (read frequency = 14%–18%) and three homoplasmic variants initially missed by manual inspection or not identified due to a sequence gap. When we included the four heteroplasmic concordant variants, our results suggest that 454 sequencing may detect heteroplasmy at the level reliably detected by Sanger sequencing (above ∼20%) [26] but also below that limit [14].

Of the two heteroplasmic variants missed by Sanger analysis, 7501T>C is a novel, potential mutation in the mtDNA tRNA serine 1 gene (*MT-TS1*). Mutations in tRNA serine 1 have been described in patients with mitochondrial disease [27] but no one with cardiomyopathy or sudden cardiac death as for our patient (Case 5). However, although 7501T>C was not found in the mtDNA databases with more than 5,000 sequences [28], [29], it is possible that 7501T>C is only a benign polymorphism not found in the specific mtDNA haplogroups comprising the databases. As another possible explanation, 7501T>C may be one of many heteroplasmic variants detectable in normal individuals at low levels [14]. Therefore, we emphasize that we cannot conclude that 7501T>C is truly a mutation until we obtain additional evidence through family studies, functional studies of 7501T>C for mitochondrial defects and population studies to investigate the amount of normal mtDNA variation within this patient's haplogroup.

### Sequence coverage needed to detect mtDNA variants

In addition to our 454 and Sanger comparisons, we used the deep 454 sequence coverage (average 1300×) and computer simulations to estimate the minimum amounts of coverage needed to detect homoplasmic and heteroplasmic mtDNA variants. The results suggest that 20× coverage was needed for homoplasmy and at least 200× coverage for heteroplasmy greater than 10%, the limit of this study. This is consistent with the observed 10-20× coverage sufficient for the detection of homoplasmy in a previous 454 study of 14 mtDNAs [13] and the ≥200× coverage selected as criteria for potential heteroplasmy in an Illumina GAII sequencing study of three mtDNAs [16]. Thus, detection of heteroplasmic mtDNA variants at frequencies above 10% requires greater 454 coverage than the >30× coverage estimated for the detection of heterozygous nDNA variants [21], [30], and detection of heteroplasmic variants at lower frequencies may require deeper sequencing, coverages estimated to be at least 1,500× for ≥5% [15] and 15,000× to 100,000× for as low as 2% [14], [31].

In summary, our results support the potential use of 454 sequencing for detection of mtDNA substitutions with heteroplasmy of greater than 10%. Using this data, we also estimate a single 454 GS FLX run would obtain sufficient coverage (20×) to detect homoplasmic mtDNA variants in 300 mtDNAs. These results, thus, support high-throughput population studies on mtDNA variation as another potential use of 454 sequencing [12], [13]. As the performance and output for sequencing technologies continue to improve, we may anticipate higher accuracies and deeper coverages that will enable low cost global analysis of mtDNA variation and highly sensitive mtDNA mutation detection. We hope that this will identify key genetic determinants for heart failure, cardiomyopathy and mitochondrial disease leading to the personalized understanding of disease mechanisms and personalized treatments.

## Materials and Methods

### Study population/ethics statement

We evaluated 20 patients referred to Dr. Eloisa Arbustini at Center for Inherited Cardiovascular Diseases in Pavia, Italy (Table 1). This included 18 patients with mitochondrial cardiomyopathy and two suspected of mitochondrial disease. The 20 cases were a subset of 29 cases to be reported on a web-based bioinformatics approach to identify potential mtDNA mutations by Sanger sequencing (unpublished data). Case 1 was from previously reported HCM family with an *MYH7* mutation and possible mtDNA mutation 9957T>C [17]. We obtained informed written consent of all participants; the study was approved by the bioethics committee of the IRCCS Foundation Policlinico San Matteo in Pavia and by the UC Irvine Institutional Review Board for de-identified DNA samples (#2009-6931).

### Mitochondrial DNA sequencing

We extracted genomic DNA from blood except for Case 7 in which we used heart tissue. Sanger sequencing method (costs ∼$130 per mtDNA) and mtDNA data analyses are described in a separate report (unpublished data).

**Figure 5:**
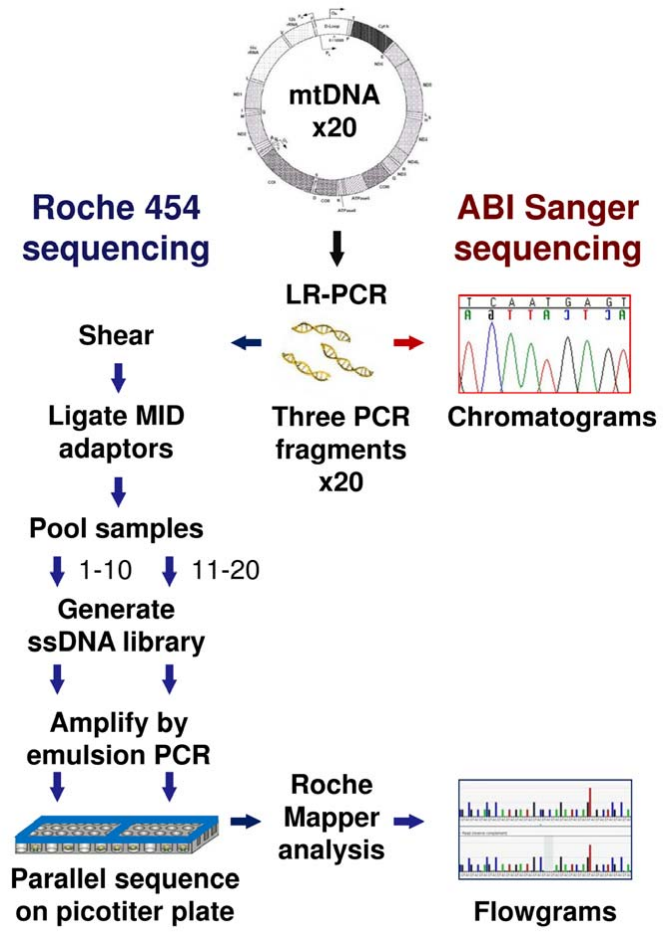
Mitochondrial DNA sequencing approach. Schematic depicts steps in ABI Sanger and Roche 454 sequencing [23]. We isolated DNA from 20 cases and amplified mitochondrial DNA (mtDNA) by long range PCR (LR-PCR). We sequenced the PCR fragments using standard Sanger and 454 sequencing methods. For 454, the fragments were sheared and then ligated to Multiplex Identifier (MID) adaptors. Next, we made two pooled samples by combining the tagged fragments for cases 1–10 and cases 11–20. Single-stranded DNA (ssDNA) libraries were made from each pooled sample and clonally amplified by emulsion PCR. Parallel sequencing was done on a single picotiter plate divide into two regions. The final data, represented as a Sanger chromatogram and 454 flowgrams, are shown.

For Roche 454 sequencing (Figure 5), we first PCR amplified mtDNA from genomic DNA using Roche Expand Long Range PCR dNTPack (Roche Applied Science, Indianapolis, IN). Using three pairs of mtDNA-specific primers, we produced three overlapping PCR fragments; A: 6929 bp, position 569–7497; B: 7050 bp, position 5061–12111; C: 6866 bp, 11107–1405 (Figure 1). We purified each fragment using Roche High Pure PCR Product Purification Kit. Primer sequences and PCR conditions are available upon request.

We conducted Roche 454 FLX sequencing for the 20 cases at the Genotyping & Sequencing Core/Orphan Disease Testing Center at the University of California, Los Angeles. Using standard protocols [23], we “shotgun” sequenced the three mtDNA PCR fragments for each of the 20 cases (costs ∼$835 per mtDNA). First, the three fragments were quantified by fluorimetry using the PicoGreen Assay, and 2 micrograms of each fragment were combined. Each of the 20 resulting samples was sheared using the Covaris system (Covaris, Inc., Woburn, MA) and the sizes of the DNA fragments were checked using the Agilent 2100 Bioanalyzer system (Agilent Technologies, Inc., Santa Clara, CA). Roche's 10-base Multiplex Identifier (MID) Adaptors were added, and equimolar quantities of the tagged fragments for the first 10 cases and the last 10 cases were pooled together. For each of the two pooled samples, single-stranded DNA libraries were made and amplified by emulsion PCR. We conducted a single 454 sequencing run using one PicoTiterPlate divided into two regions, each with 10 MID tagged samples.

### Bioinformatics analysis

We used the Roche 454 GS Mapper software (version 2.0.01) to assemble and compare the sequencing reads to the mtDNA reference sequence [GenBank: NC_012920] [32]. After standard image and signal processing, we sorted the sequencing reads from the Standard Flowgram Format (SFF) files using the distinct MID for each case. Reads were mapped to the reference sequence which produced a consensus sequence and identified “high-confidence differences” (HCDiffs). The criteria for HCDiffs were defined by GS Mapper: variants detected in at least three unique (non-duplicate) sequencing reads of high quality with both forward and reverse reads and found in >10% of the total unique sequencing reads.

We categorized each HCDiff by the variant type (single nucleotide substitution, insertion/deletion (indel) or repeat polymorphism) and by the presence or absence of heteroplasmy. HCDiffs at positions 303–315, 522–523, 574, 16180–16193 and 16519 were considered repeat polymorphisms [33], [34]. For assessment of heteroplasmy, we developed criteria based on variant frequency, the number of variant reads divided by the total number of unique sequencing reads. We also carefully reviewed the variant and non-variant reads in the HCDiff text files to identify results most likely due to homopolymers, a known false positive source because of the technical limitations in 454 pyrosequencing [11], [23]–[25]. We classified an HCDiff as heteroplasmic if: (1) the variant frequency was between 0.10 and 0.80 and (2) all non-variant reads contained only the reference allele, and as homoplasmic if: (1) variant frequency was ≥0.80 and (2) all non-variant reads may be explained by a homopolymer error that occurred at or adjacent to the nucleotide position. Since a variant had to be detected in >10% of unique reads to be considered an HCDiff, we did not evaluate variants with ≤0.10 frequencies.

All new sequence data for the 20 cases has been deposited in GenBank [accession numbers HM765456-HM765475].

### Simulation studies

To estimate the minimal coverage for variant detection, we calculated the detection rate at different levels of simulated read coverage. We estimated from an average read length of ∼375 bases that 1× coverage of the ∼16.5 kb mitochondrial sequence would be ∼50 reads. Therefore, to simulate nine coverage levels (2×, 5×, 10×, 20×, 30×, 50×, 100×, 300× and 500×), we generated random subsamples of 100, 250, 500, 1000, 1500, 2500, 5000, 15000 and 25000 sequencing reads, respectively for each of the 20 cases. Three simulations were done for each coverage level. As before, we used Roche's GS Mapper to call HCDiffs and then filtered for single nucleotide polymorphisms. For each coverage level, we calculated the variant detection rate, the proportion of HCDiffs called out of the total number variants detected with full coverage.

### Performance metrics and statistical analysis

To compare the mtDNA sequencing performances of the 454 and Sanger platforms, we used defined metrics [11]. We used JMP 7.0 (SAS Institute, Cary, NC) for statistical analysis and graphs. We considered p<0.05 as a significant difference.

To calculate performance metrics, we defined annotations that represented genotype differences between 454 and Sanger platforms [11] but modified to take heteroplasmy into account for mtDNA analysis (Table S3). For this analysis, we excluded the results for 31 nucleotide positions that had repeat polymorphisms: 303–315, 522–523, 574, 16180–16193 and 16519. When multiple Sanger sequence peaks are found staggered at these positions, it is unclear whether this finding resulted from true heteroplasmy or possibly an artifact due to polymerase error [20], [35].

For 454 sequencing, we calculated sequencing and variant accuracies (Table S3). We defined sequencing accuracy (SA) as the percentage of the genotype calls that were concordant between 454 and Sanger vs. the sum of numbers of concordant and discordant calls; it can be also expressed as the symbols defined in Table S3. SA  =  (A1 + B2 + C3) / (A1 + A2 + A3 + B1 + B2 + B3 + C1 + C2 + C3). We defined variant accuracy (VA) as the percentage of homoplasmic and heteroplasmic variant calls by Sanger that were concordant with 454 vs. total number of variants found by both technologies; we calculated variant accuracy as VA  =  (B2 + C3) / (A2 + A3 + B2 + B3 + C2 + C3).

Finally, we calculated the false positive and false negative rates for both platforms (Table S3). The false positive rates were the proportion of reference genotypes (i.e., confirmed genotypes with false positive or true negative results) that were called as variants by each sequencing platform. The false negative rates were the proportion of variant genotypes (i.e., confirmed genotypes with false negative or true positive results) that were not called as variants by each sequencing platform.

## Supporting Information

Table S1 Mitochondrial DNA variants identified by 454 and/or Sanger sequencing, n = 614.
(0.16 MB XLS)

Table S2 454 Mapping Statistics.
(0.02 MB XLS)

Table S3 Sequencing performance metrics: data tables (A) and calculations (B).
(0.02 MB XLS)

Table S4 Coverage simulations for heteroplasmic and homoplasmic mtDNA substitutions.
(0.08 MB XLS)
